# Platinum-Based Drugs Cause Mitochondrial Dysfunction in Cultured Dorsal Root Ganglion Neurons

**DOI:** 10.3390/ijms21228636

**Published:** 2020-11-16

**Authors:** Markus Leo, Linda-Isabell Schmitt, Patricia Küsterarent, Andrea Kutritz, Tienush Rassaf, Christoph Kleinschnitz, Ulrike B. Hendgen-Cotta, Tim Hagenacker

**Affiliations:** 1 Department of Neurology, NeuroScienceLab, Medical Faculty, University Medicine Essen, 45147 Essen, Germany; Linda-Isabell.Schmitt@UK-Essen.de (L.-I.S.); Patricia.Kuesterarent@rub.de (P.K.); Andrea.Kutritz@UK-Essen.de (A.K.); Christoph.Kleinschnitz@UK-Essen.de (C.K.); Tim.Hagenacker@UK-Essen.de (T.H.); 2 Department of Cardiology and Vascular Medicine, CardioScienceLabs, University Medicine Essen, Medical Faculty, 45147 Essen, Germany; Tienush.Rassaf@UK-Essen.de (T.R.); Ulrike.Hendgen-Cotta@UK-Essen.de (U.B.H.-C.)

**Keywords:** dorsal root ganglia, neuropathy, oxaliplatin, cisplatin, ROS, mitochondrial stress, peripheral nervous system, chemotherapy, TRP channels, neuropathic pain

## Abstract

Cisplatin and oxaliplatin are treatment options for a variety of cancer types. While highly efficient in killing cancer cells, both chemotherapeutics cause severe side effects, e.g., peripheral neuropathies. Using a cell viability assay, a mitochondrial stress assay, and live-cell imaging, the effects of cis- or oxaliplatin on the mitochondrial function, reactive oxygen species (ROS) production, and mitochondrial and cytosolic calcium concentration of transient receptor potential ankyrin 1 (TRPA1)- or vanilloid 1 (TRPV1)-positive dorsal root ganglion (DRG) neurons of adult Wistar rats were determined. Mitochondrial functions were impaired after exposure to cis- or oxaliplatin by mitochondrial respiratory chain complex I-III inhibition. The basal respiration, spare respiratory capacity, and the adenosine triphosphate (ATP)-linked respiration were decreased after exposure to 10 µM cis- or oxaliplatin. The ROS production showed an immediate increase, and after reaching the peak, ROS production dropped. Calcium imaging showed an increase in the cytosolic calcium concentration during exposure to 10 µM cis- or oxaliplatin in TRPA1- or TRPV1-positive DRG neurons while the mitochondrial calcium concentration continuously decreased. Our data demonstrate a significant effect of cis- and oxaliplatin on mitochondrial function as an early event of platinum-based drug exposure, suggesting mitochondria as a potential target for preventing chemotherapy-induced neuropathy.

## 1. Introduction

Cytostatic drugs as cis- and oxaliplatin are still common in tumor therapy today. While cisplatin is used for the treatment of lung, testicular, or ovarian cancer, oxaliplatin is majorly used for colorectal cancer [1,2,3,4]. Beside their high efficiency in killing cancer cells, those chemotherapeutics have severe side effects like peripheral neuropathies with or without neuropathic pain, described as chemotherapy-induced peripheral neuropathy (CIPN) [5]. Cisplatin and oxaliplatin dose-dependently cause CIPN in up to 90% of patients [6,7,8,9,10]. Both chemotherapeutics form adducts with the mitochondrial DNA and consequently inhibit the replication and transcription to proteins leading to abnormalities within the dorsal root ganglion neurons (DRG neurons) and, in the end, lead to a progressive energy deficiency [11,12].

DRG neurons are cells of the peripheral nervous system responsible for the detection of noxious (potentially damaging) stimuli. Therefore, these neurons express a specialized setup of receptors and channels that can be activated by mechanical, thermal, or chemical stimuli and contribute to typical symptoms of CIPN as hyperalgesia, allodynia, or numbness ([13,14,15]. Several of these receptors or channels such as voltage-gated calcium channels or type 1 lysophosphatidic acid (LPA_1_) receptors are discussed as playing a critical role in the induction of polyneuropathies or neuropathic pain in different models ([16,17,18,19,20]. Several cation channels facilitate nociception, e.g., the transient receptor potential channels (TRP), a family of cation channels that are crucial transducers of nociceptive stimuli contributing to sensory input. The transient receptor potential ankyrin (TRPA) present in mammals is TRPA1. It reacts to noxious cold (<17 °C) stimuli as well as chemical substances like allicin, cinnamaldehyde, and isothiocyanates [21]. TRPA1 knockout mice showed little to no reactions when in contact with reactive chemicals, e.g., formalin or tear gas [22]. Prior studies suggest TRPA1 as a potential target for pain therapy [23].

The vanilloid receptor 1 (TRPV1) activates at high temperatures (>43 °C) by capsaicin and an acidic pH [24]. TRPV1 also plays a crucial role in inflammation-related pain. By tissue damage, TRPV1 channels are sensitized to noxious stimuli by releasing inflammatory mediators, causing hyperalgesia and allodynia [25].

Next to damage in the deoxyribonucleic acid (DNA) of DRG neurons, mitochondrial and dysfunctional changes in the calcium homeostasis and increased reactive oxygen species (ROS) have been suggested to cause CIPN [12,26,27,28,29,30].

Dysfunction and changes in expression of voltage-gated calcium channels (VGCC) in DRG neurons have been shown to contribute to calcium-dyshomeostasis after cis- or- oxaliplatin exposure [16,17,18,31].

Using a mitochondrial stress assay, live-cell antibody staining, and imaging, the influence of cisplatin or oxaliplatin on the mitochondrial function of DRG neurons was examined.

## 2. Results

### 2.1. Viability of DRG Neurons Is Not Changed by Small Concentrations of Cis- or Oxaliplatin after 24 h

A luminescence viability assay was performed to examine the influence of cis- or oxaliplatin on DRG neurons’ viability. DRG neurons were cultured and exposed to 0.1 µM, 1 µM, 10 µM, or 100 µM for 24 h. As a positive control, neurons were exposed to 0.01% Triton X-100 to induce cell death after 24 h.

**Figure 1:**
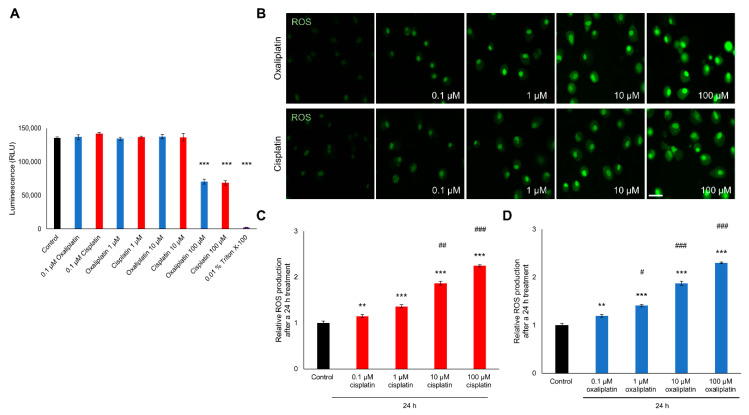
Viability and reactive oxygen species (ROS) production of cultured dorsal root ganglion (DRG) neurons after exposure to different concentrations of cis- or oxaliplatin for 24 h. (**A**) Viability of dorsal root ganglion (DRG) neurons was not affected by cis- or oxaliplatin exposure in a concentration of 0.1 µM, 1 µM, or 10 µM for 24 h, compared to untreated control neurons (*p* > 0.05). Exposure to 100 µM cis- or oxaliplatin or to 0.01% Triton X-100 reduced viability of DRG neurons after 24 h (*** *p* < 0.001). (**B**) Fluorescence of CellRox (green) in DRG neurons exposed to different concentrations of cis- or oxaliplatin for 24 h. (**C**) Reactive oxygen species (ROS) production of DRG neurons was increased by cisplatin exposure after 24 h (** *p* < 0.01, *** *p* < 0.001), compared to untreated control neurons. A concentration-dependent effect was observed for the range of 1 µM to 100 µM (^##^ *p* < 0.01, ^###^ *p* < 0.001). (**D**) Exposure of DRG neurons to different concentrations of oxaliplatin increased ROS production as well (** *p* < 0.01, *** *p* < 0.001). A concentration-dependent effect was observed for the range of 0.1 µM–100 µM (^#^ *p* < 0.05, ^###^ *p* < 0.001). *n* ≥ 200 cells per condition for ROS measurement. *n* = 3 independent experiments per condition. * = significant effect compared to control condition. ^#^ = significant effect to previous concentration. Scale: 50 µm.

When cultured DRG neurons were exposed to 0.1 µM, 1 µM, or 10 µM of cisplatin no change in viability was observed after 24 h (0.1 µM: 141850 ± 2184.98 RLU; 1 µM: 136350 ± 1619.92 RLU; 10 µM: 136650 ± 5899.78 RLU), compared to untreated control cells (135700 ± 1603.12). Exposure to 100 µM led to a decrease of the neuronal viability (68450 ± 3529.04 RLU, *** *p* < 0.001) (Figure 1A).

Similar results were observed when DRG neurons were exposed to 0.1 µM, 1 µM or 10 µM oxaliplatin. Furthermore, here no effect on cell viability was shown (0.1 µM: 136850 ± 3666.17 RLU; 1 µM: 134350 ± 2146.50 RLU; 10 µM: 137550 ± 3245.63 RLU). In contrast to these findings, exposure to 100 µM oxaliplatin resulted in a decreased viability (100 µM: 70440 ± 3420.61; *** *p* < 0.001). Furthermore, exposure to 0.01% Triton-X100 led to a decreased viability of cultured DRG neurons (2256 ± 43.41 RLU; *** *p* < 0.001) (Figure 1A).

### 2.2. ROS Production Is Increased in DRG Neurons after Cis- or Oxaliplatin Treatment for 24 h

To measure the influence of chemotherapeutics on ROS production in DRG neurons, cell were cultured as described above and exposed to 0.1 µM, 1 µM, 10 µM, or 100 µM cis- or oxaliplatin for 24 h and stained with CellRox (Figure 1B).

When DRG neurons were exposed to different concentrations of cisplatin, ROS production was increased after 24 h (0.1 µM: 1.14 ± 0.03, ** *p* < 0.01; 1 µM: 1.36 ± 0.03, *** *p* < 0.001; 10 µM: 1.86 ± 0.04, *** *p* < 0.001; 100 µM: 2.24 ± 0.02, *** *p* < 0.001) compared to untreated control neurons (1 ± 0.03). A concentration-dependent effect was observed for a concentration range of 1 µM to 100 µM (^##^ *p* < 0.01, ^###^ *p* < 0.001) (Figure 1C).

Exposure of DRG neurons to different concentrations of oxaliplatin led to similar results, also here the ROS productions was increased (0.1 µM: 1.19 ± 0.02, ** *p* < 0.01; 1 µM: 1.41 ± 0.02; *** *p* < 0.001; 10 µM: 1.87 ± 0.04, *** *p* < 0.001; 100 µM: 2.30 ± 0.01; *** *p* < 0.001). A concentration-depend effect was shown for the range of 0.1 µM to 100 µM (^#^ *p* < 0.05, ^###^ *p* < 0.001) (Figure 1D).

### 2.3. Cis- and Oxaliplatin Induce Mitochondrial Stress in DRG Neurons

A mitochondrial stress assay was conducted to determine the different respiration rates during the addition of Oligomycin, carbonyl cyanide-4 (trifluoromethoxy)-phenylhydrazone (FCCP), and Rotenone and Antimycin A. Cells were exposed either 10 µM cis- or oxaliplatin overnight.

**Figure 2:**
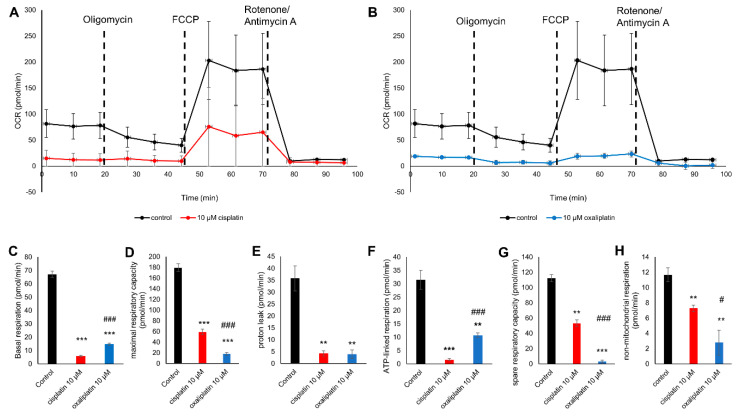
Mitochondrial function after an exposure of 10 µM cis- and oxaliplatin to cultured sensory neurons. (**A**) Oxygen consumption rate (OCR) during the mitochondrial stress assay of sensory neurons exposed to 10 µM cisplatin for 24 h compared to a control. (**B**) OCR during the mitochondrial stress assay of the sensory neurons exposed to 10 µM oxaliplatin for 24 h compared to a control. (**C**) The basal respiration (BR) rate dropped after exposure to cisplatin to 5.856 ± 0.710 pmol/min (*** *p* < 0.001) and oxaliplatin to 14.682 ± 0.987 pmol/min (*** *p* < 0.001) compared to the control at 67.095 ± 2.433 pmol/min. Cisplatin-exposed DRG neurons also showed lower BR than oxaliplatin-exposed DRG neurons (^###^ *p* < 0.001). (**D**) The maximal respiratory capacity (MRC) decreased after exposure to cisplatin at 59.252 ± 4.806 pmol/min (*** *p* < 0.001) and oxaliplatin at 17.857 ± 2.594 (*** *p* < 0.001) compared to the control cells at 179.528 ± 6.965 pmol/min. The DRG neurons exposed to oxaliplatin showed a lower MRC than cisplatin-exposed DRG neurons (^###^ *p* < 0.001). (**E**) The proton leak (PL) is reduced to 4.241 ± 1.105 pmol/min (** *p* < 0.01) after exposure to cisplatin and at 3.922 ± 1.748 pmol/min (** *p* < 0.01) to oxaliplatin compared to 35.776 ± 5.207 pmol/min for the non-treated cells. No difference in the effect of cis- or oxaliplatin exposure could be observed (*p* > 0.05) (**F**) The adenosine triphosphate (ATP)-linked respiration (ATPR) was determined at 31.521 ± 3.503 pmol/min at the control and is decreased after exposure to cisplatin at 1.615 ± 0.403 pmol/min (*** *p* < 0.001) and 10.760 ± 0.763 pmol/min (** *p* < 0.01) after exposing the cells to oxaliplatin. Cisplatin-exposed DRG neurons show lower ATPR than oxaliplatin-exposed DRG neurons (^###^ *p* < 0.001). (**G**) The spare respiratory capacity (SRC) was decreased after exposure to cisplatin at 53.396 ± 4.138 pmol/min (** *p* < 0.01) and oxaliplatin at 3.175 ± 1.976 (*** *p* < 0.001) pmol/min in comparison to 112.433 ± 4.570 pmol/min at the control. Oxaliplatin-exposed DRG neurons had a lower SRC than cisplatin-exposed DRG neurons (^###^ *p* < 0.001). (**H**) The non-mitochondrial respiration (NMR) is decreased after exposure to cisplatin at 7.294 ± 0.432 pmol/min (** *p* < 0.01) and oxaliplatin at 2.786 ± 1.633 pmol/min (** *p* < 0.01) compared to 11.672 ± 0.913 pmol/min for the control cells. Oxaliplatin-exposed DRG neurons showed lower NMR than cisplatin-exposed DRG neurons (^#^ *p* < 0.05). *n* = 3 independent experiments per condition. * = significant effect compared to control condition. ^#^ = significant effect between cis- and oxaliplatin.

OCR was lower for the sensory neurons exposed to 10 µM cisplatin or oxaliplatin than in untreated cells (* *p* < 0.05) (Figure 2A,B).

The basal respiration (BR) rate decreased after cisplatin exposure to 5.85 ± 0.71 pmol/min (*** *p* < 0.001) and after oxaliplatin exposure to 14.68 ± 0.98 pmol/min (*** *p* < 0.001) compared to the control at 67.09 ± 2.43 pmol/min. Cisplatin exposure had a more substantial effect on the BR than oxaliplatin (^###^ *p* < 0.001) (Figure 2C). As well as the BR, the maximal respiratory capacity (MRC) also decreased after a cisplatin exposure to 59.25 ± 4.80 pmol/min (*** *p* < 0.001) and after an oxaliplatin exposure to 17.857 ± 2.594 compared to the control cells at 179.52 ± 6.96 pmol/min (*** *p* < 0.001). Oxaliplatin exposure had a more substantial effect on the MRC than cisplatin (^###^ *p* < 0.001) (Figure 2D). Under control conditions, proton leak (PL) was 35.77 ± 5.20 pmol/min. After cisplatin exposure, PL decreased to 4.24 ± 1.10 pmol/min (** *p* < 0.01) and was reduced to 3.92 ± 1.74 pmol/min after an oxaliplatin exposure (** *p* < 0.01). There was no difference in PL between cis-or oxaliplatin (*p* > 0.05) (Figure 2E).

The adenosine triphosphate-linked respiration (ATPR) is 31.52 ± 3.50 pmol/min at the control level, whereas it was decreased after exposure to cisplatin to 1.61 ± 0.40 pmol/min (*** *p* < 0.001) and to 10.76 ± 0.76 pmol/min (** *p* < 0.01) after exposing the cells to oxaliplatin. Cisplatin exposure had a more substantial effect on the ATPR than oxaliplatin (^###^ *p* < 0.001) (Figure 2F). The spare respiration capacity (SRC) was also decreased after exposure with cisplatin for 24 h to 53.39 ± 4.13 pmol/min (** *p* < 0.01) compared to control conditions (112.43 ± 4.57 pmol/min). Exposure with oxaliplatin for 24 h reduced the SRC to 3.17 ± 1.97 pmol/min (*** *p* < 0.001). Oxaliplatin exposure had a more substantial effect on the SRC than cisplatin (^###^ *p* < 0.001) (Figure 2G).

Under control conditions, non-mitochondrial respiration (NMR) was 11.67 ± 0.91 pmol/min. Here, an exposure with cis- or oxaliplatin for 24 h decreased the non-mitochondrial respiration (cisplatin: 7.29 ± 0.43 pmol/min (** *p* < 0.01) and oxaliplatin: 2.78 ± 1.63 pmol/min (** *p* < 0.01)). Oxaliplatin exposure had a stronger effect on the non-mitochondrial respiration than cisplatin (^#^ *p* < 0.05) (Figure 2H).

### 2.4. Change in Cytosolic and Mitochondrial Calcium Concentrations of TRPA1- or TRPV1-Positive DRG Neurons Is Caused by Exposure to Cisplatin

**Figure 3:**
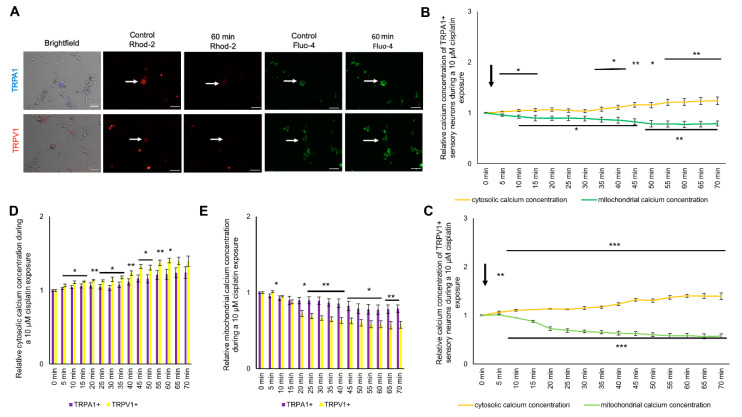
Cytosolic and mitochondrial calcium concentration of TRPA1- or TRPV1-positive DRG neurons during exposure to 10 µM cisplatin. (**A**) Immunostaining of transient receptor potential ankyrin 1 (TRPA1)- or transient receptor potential vanilloid 1 (TRPV1)-positive DRG neurons and live imaging of Fluo-4 (green) and Rhod-2 (red). (**B**) The cytosolic calcium of TRPV1-positive DRG neurons concentration instantly increased to 1.067 ± 0.018 (* *p* < 0.05) after 5 min and continuously increased to 1.394 ± 0.068 (** *p* < 0.01) after 70 min. The mitochondrial calcium concentration declined to 1.095 ± 0.009 (* *p* < 0.05) after 5 min for TRPV1-positive DRG neurons and steadily declined to 0.577 ± 0.044 after 70 min (** *p* < 0.01). (**C**) During the exposure of 10 µM cisplatin, the cytosolic calcium concentration of TRPA1-positive neurons increased after 5 min to 1.024 ± 0.011 (** *p* < 0.01) until 70 min at 1.240 ± 0.074 (*** *p* < 0.001). The mitochondrial calcium concentration was lower after 10 min at 0.928 ± 0.029 (*** *p* < 0.001) until it reached 0.790 ± 0.050 at 70 min (*** *p* < 0.001). (**D**) The cytosolic calcium concentration of TRPV1-positive DRG neurons was increased after 15 min to 65 min compared to TRPA1-positive DRG neurons (* *p* < 0.05, ** *p* < 0.01). (**E**) The mitochondrial calcium concentration of TRPA1- or TRPV1-positive DRG neurons was different after 5 min until the end of the experiment (* *p* < 0.05, ** *p* < 0.01). TRPV1-positive sensory neurons had a lower mitochondrial calcium concentration during exposure to 10 µM cisplatin after 15 min (* *p* < 0.05, ** *p* < 0.01). *n* = 6 cells per condition. * = significant effect between TRPA1+ and TRPV1+ DRG neurons. Scale = 50 µm.

To determine the effect of cisplatin on the cytosolic and mitochondrial calcium concentration in TRPA1- or TRPV1-positive sensory neurons, cells were cultured and stained as described above. Cisplatin was applied directly while measuring, and cells were observed over 70 min (Figure 3A). After exposure to 10 µM cisplatin, cytosolic calcium was increased in TRPV1-positive neurons. The cytosolic calcium steadily increased to a maximum of 1.39 ± 0.05 (** *p* < 0.01) at 70 min. The mitochondrial calcium concentration dropped below the control level after 10 min (* *p* < 0.05) and continuously decreased to 0.56 ± 0.05 (** *p* < 0.01) after 70 min (Figure 3B). The cytosolic calcium concentration of TRPA1-positive neurons increased to 1.05 ± 0.02 (*** *p* < 0.001) after 15 min with a maximum at 1.24 ± 0.07 (*** *p* < 0.001) after 70 min. The intramitochondrial calcium concentration continuously decreased to a minimum at 70 min (0.79 ± 0.05, *** *p* < 0.001) (Figure 3C).

Comparing the increase of the cytosolic calcium concentration between TRPA1- and TRPV1-positive cells after cisplatin application, the cytosolic calcium concentration of TRPV1-positive neurons was more robustly increased (5 min until 60 min) compared to TRPA1-positive neurons (* *p* < 0.05, ** *p* < 0.01). The mitochondrial calcium concentration also had a stronger decrease in TRPV1-positive neurons after 5 min (* *p* < 0.05, ** *p* < 0.01) (Figure 3D,E).

### 2.5. Exposure to Oxaliplatin Changes Cytosolic and Mitochondrial Calcium Concentrations of TRPA1- or TRPV1-Positive DRG Neurons

**Figure 4:**
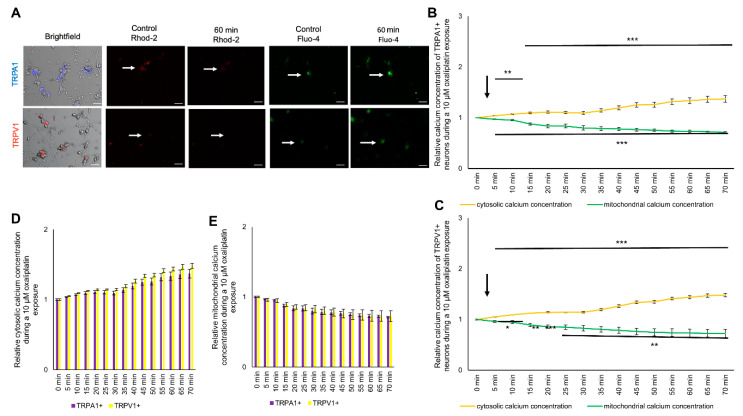
Cytosolic and mitochondrial calcium concentration of TRPA1- or TRPV1-positive DRG neurons during exposure to 10 µM oxaliplatin. (**A**) Immunostaining of TRPA1- or TRPV1-positive DRG neurons and live imaging of Fluo-4 (green) and Rhod-2 (red) before and after application of 10 µM cisplatin or oxaliplatin. (**B**) TRPA1-positive DRG neurons showed an immediate increase of the cytosolic calcium concentration to 1.037 ± 0.006 (** *p* < 0.01) after 5 min and continuously increased to 1.372 ± 0.062 after 70 min (*** *p* < 0.001). The mitochondrial calcium concentration decreased after 5 min to 0.967 ± 0.008 (*** *p* < 0.001) and declined to 0.712 ± 0.010 after 70 min (*** *p* < 0.001). (**C**) The TRPV1-positive DRG neurons showed an immediate increase of the cytosolic calcium concentration to 1.049 ± 0.005 (*** *p* < 0.001) after 5 min and increased to 1.480 ± 0.034 after 70 min (*** *p* < 0.001). The mitochondrial calcium concentration decreased to 0.959 ± 0.020 (* *p* < 0.05) after 5 min and to 0.725 ± 0.077 (** *p* < 0.01) after 70 min (* *p* < 0.05). (**D**) No difference could be determined in the cytosolic calcium concentration of TRPA1- or TRPV1-positive DRG neurons (*p* > 0.05). (**E**) No difference could be determined in the mitochondrial calcium concentration of TRPA1- or TRPV1-positive DRG neurons (*p* > 0.05). *n* = 6 cells per condition. * = significant effect between TRPA1+ and TRPV1+ DRG neurons. Scale = 50 µm.

To determine the effect of oxaliplatin on the cytosolic and mitochondrial calcium concentration in TRPA1- or TRPV1-positive sensory neurons, cells were cultured and stained as described above. Cisplatin was applied directly while measuring, and cells were observed over 70 min (Figure 4A). Both subpopulations showed an immediate reaction to cisplatin and oxaliplatin. During an exposure with 10 µM oxaliplatin, the relative cytosolic calcium concentration increased continuously to a maximum for TRPA1-positive neurons at 1.37 ± 0.06 (*** *p* < 0.001) and a maximum for TRPV1-positive neurons at 1.48 ± 0.03 (*** *p* < 0.001) after 70 min (Figure 4B,C). The mitochondrial calcium concentration continuously decreased to 0.71 ± 0.01 (*** *p* < 0.001) for TRPA1 and to 0.72 ± 0.07 (** *p* < 0.01) for TRPV1-positive neurons after 70 min (Figure 4B,C). The increase of the cytosolic calcium concentration and the decrease of the mitochondrial calcium concentration was not different in TRPA1- or TRPV1-positive neurons (*p* > 0.05) (Figure 4D,E).

### 2.6. Exposure to Cis- or Oxaliplatin Results in Differences in Cytosolic and Mitochondrial Calcium in TRPA1- or TRPV1-Positive DRG Neurons

**Figure 5:**
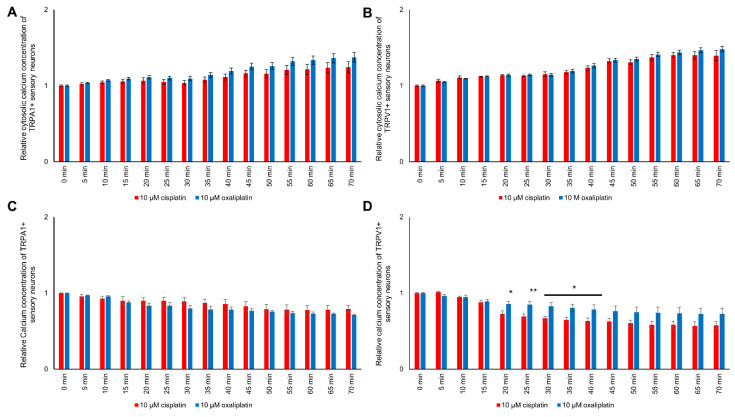
Relative cytosolic and mitochondrial calcium concentration during exposure to 10 µM cis- or oxaliplatin of TRPA1- or TRPV1-positive DRG neurons. (**A**) No differences in the cytosolic calcium concentration of TRPA1-positive DRG neurons after exposure to 10 µM cis- or oxaliplatin could be determined (*p* > 0.05). (**B**) The cytosolic calcium of TRPV1-positive DRG neurons concentration during exposure to cis- or oxaliplatin showed no difference (*p* > 0.05). (**C**) During exposure to 10 µM cis- or oxaliplatin, no difference in the mitochondrial calcium concentration of TRPA1-positive DRG neurons could be determined (*p* > 0.05). (**D**) While exposing TRPV1-positive DRG neurons, differences between the two chemotherapeutics on the mitochondrial calcium concentration could be determined after 20 min. The mitochondrial calcium concentration of TRPV1-positive DRG neurons exposed to oxaliplatin was higher at 0.856 ± 0.033 compared to 0.727 ± 0.040 (* *p* < 0.05). The mitochondrial calcium concentration was higher for oxaliplatin-exposed TRPV1-positive DRG neurons until 0.786 ± 0.058 after 40 min (* *p* < 0.05). *n* = 6 cells per condition. * = significant effect between cis- and oxaliplatin, ** *p* < 0.01

Comparing the influences of cis- or oxaliplatin on the cytosolic and mitochondrial calcium concentration in TRPA1- or TRPV1-positive DRG neurons, no differences could be determined (*p* > 0.05) (Figure 5A–C).

However, differences in cis- or oxaliplatin’s effects on mitochondrial calcium concentration of TRPV1-positive DRG neurons could be measured. In TRPV1-positive neurons, the mitochondrial calcium concentration was lower after exposure to cisplatin than to oxaliplatin between 20 min and 40 min (* *p* < 0.05, ** *p* < 0.01) (Figure 5D).

### 2.7. Modulation of ROS Production in TRPA1- or TRPV1-Positive DRG Neurons after Cisplatin Exposure

**Figure 6:**
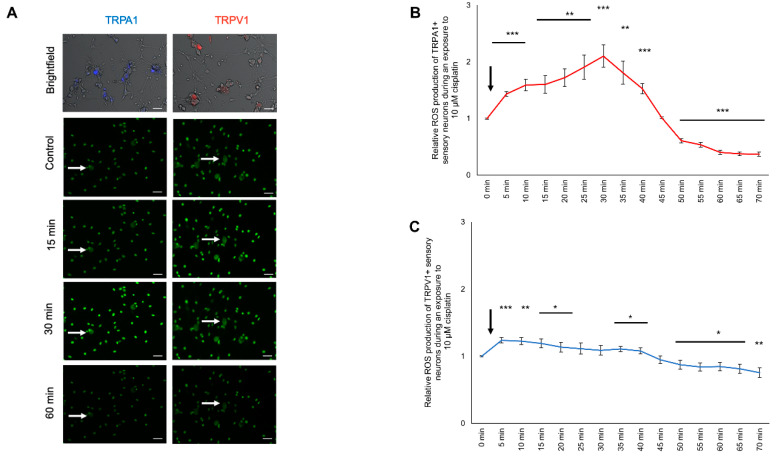
ROS production of TRPA1- or TRPV1-positive DRG neurons during exposure to 10 µM cisplatin. (**A**) Fluorescence of CellRox (green) in TRPA1- or TRPV1-positive DRG neurons during exposure to 10 µM cisplatin at different time points. (**B**) TRPA1-positive sensory neurons showed an instant increase of the ROS production to 1.431 ± 0.042 (*** *p* < 0.001) after 5 min and reached its peak at 2.100 ± 0.197 after 30 min (*** *p* < 0.001). After 50 min, the ROS production dropped below the control level at 50 min to 0.607 ± 0.036 (*** *p* < 0.001) and further declined to 0.370 ± 0.035 (*** *p* < 0.001) after 70 min. (**C**) After exposure to 10 µM cisplatin of the TRPV1-positive DRG neurons, the ROS production increased to 1.238 ± 0.042 (*** *p* < 0.001) after 5 min and dropped below control level at 50 min at 0.873 ± 0.068 (* *p* < 0.05) and steadily declined to 0.756 ± 0.072 (** *p* < 0.01) after 70 min. *n* = 6 cells per condition. * = significant effect compared to control level. Scale = 50 µm.

To investigate the effect of cisplatin on the ROS production change in TRPA1-positive and TRPV1-positive sensory neurons, cells were cultured and stained as described above. Cisplatin (10 µM) was applied directly while measuring and was observed for 70 min (Figure 6A). The ROS production of TRPA1-positive neurons during 10 µM cisplatin exposure showed a continuous increase with a maximum at 1.80 ± 0.20 (*** *p* < 0.001) after 30 min. Afterward, it continuously decreased and reached the control level after 45 min (1.01 ± 0.01; *p* > 0.05). The relative ROS production further decreased to 0.370 ± 0.035 (*** *p* < 0.001) at 70 min (Figure 6B).

The ROS production of TRPV1-positive neurons continuously increased to a maximum of 1.23 ± 0.07 (*** *p* < 0.001) 5 min after exposure to cisplatin. The ROS production decreased after 35 min and dropped below the control level after 50 min (0.873 ± 0.068; * *p* < 0.05) with a minimum at 0.75 ± 0.07 (** *p* < 0.01) after 70 min (Figure 6C).

### 2.8. Modulation of ROS Production in TRPA1- or TRPV1-Positive DRG Neurons after Oxaliplatin Exposure

**Figure 7:**
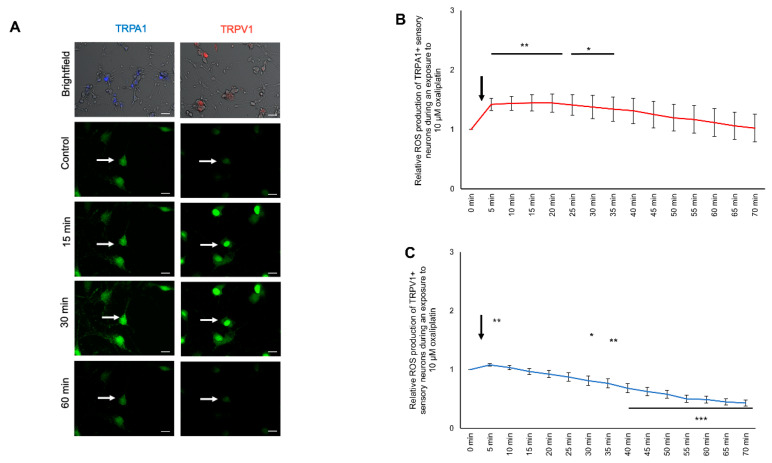
ROS production of TRPA1- or TRPV1-positive DRG neurons during exposure to 10 µM oxaliplatin. (**A**) Fluorescence of CellRox (green) of TRPA1- or TRPV1-positive DRG neurons at different time points. (**B**) The ROS production of TRPA1-positive DRG neurons increased after 5 min to 1.421 ± 0.103 (** *p* < 0.01) during exposure to 10 µM oxaliplatin and dropped to control level after 40 min at 1.31 ± 0.194 (*p* > 0.05). (**C**) The TRPV1-positive sensory neurons had an increased ROS production after 5 min at 1.080 ± 0.020 (** *p* < 0.01) and dropped to the control level after 10 min at 1.033 ± 0.036 (*p* > 0.05). After 30 min, the ROS production declined below the control level at 0.810 ± 0.079 (* *p* < 0.05) and steadily declined to 0.429 ± 0.050 after 70 min (*** *p* < 0.001). *n* = 6 cells per condition. * = significant effect compared to control level. Scale = 20 µm.

To investigate the effect of oxaliplatin on ROS production in TRPA1- and TRPV1-positive sensory neurons, cells were cultured and stained as described above. Oxaliplatin (10 µM) was applied directly while measuring and was observed over 70 min (Figure 7A). The ROS production of TRPA1-positive neurons during the oxaliplatin exposure increased after 5 min (1.42 ± 0.11; ** *p* < 0.01). It continuously increased with a peak at 15 min (1.44 ± 0.13; * *p* < 0.05). Afterward, the ROS production decreased towards control levels (70 min: 1.02 ± 0.23; *p* > 0.05) (Figure 7B).

The ROS production of TRPV1-positive neurons increased after 5 min at 1.08 ± 0.01 (* *p* < 0.05) and decreased to the control condition. After 30 min, the ROS production was decreased (0.81 ± 0.08; ** *p* < 0.01) with a minimum at 70 min (0.43 ± 0.05; *** *p* < 0.001) (Figure 7C).

### 2.9. ROS Production Is Different in TRPA1- and TRPV1-Positive DRG Neurons

**Figure 8:**
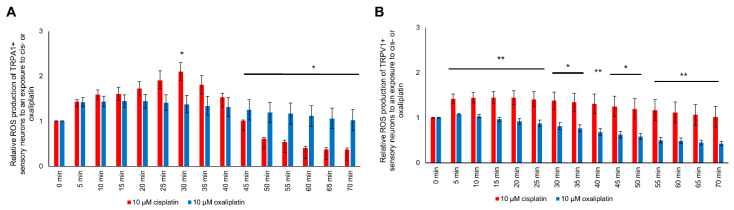
The ROS production of TRPA1- or TRPV1-positive DRG neurons during exposure to cis- or oxaliplatin. (**A**) A difference could be determined for TRPA1-positive DRG neurons at 30 min with a higher ROS production for cisplatin-exposed DRG neurons at 2.100 ± 0.197 (* *p* < 0.05). After 45 min (1.194 ± 0.223; * *p* < 0.05) until 70 min (1.022 ± 0.233; * *p* < 0.05), the ROS production was higher for oxaliplatin-exposed neurons. (**B**) The ROS production of TRPV1-positive DRG neurons was higher for cisplatin-exposed neurons over the entire experimental period (* *p* < 0.05, ** *p* < 0.01). *n* = 6 cells per condition. * = significant effect compared to control level.

The ROS production of TRPA1-positive DRG neurons after exposure to cis- or oxaliplatin was different. Cisplatin exposure had a more substantial effect on ROS production after 30 min than oxaliplatin exposure (* *p* < 0.05). After 45 min until 70 min, ROS production was decreased after cisplatin exposure compared to oxaliplatin exposure (* *p* < 0.05) (Figure 8A).

The TRPV1-positive DRG neurons showed a higher ROS production after exposure to cisplatin than oxaliplatin during the entire experimental time (* *p* < 0.05, ** *p* < 0.01) (Figure 8B).

## 3. Discussion

We here show that the platinum-based drugs cis- and oxaliplatin induce mitochondrial dysfunction of TRPA1- or TRPV1-positive DRG neurons. Here we observed changes in ROS production, mitochondrial, and cytosolic calcium concentration.

Both cisplatin and oxaliplatin are known to contribute to induction of apoptotic processes in different cell types. When cultured DRG neurons were treated with different concentrations of these chemotherapeutics, no change in cell viability was observed when cells were treated with 0.1 µM, 1 µM, or 10 µM after 24 h. Viability was only decreased by cis- or oxaliplatin in a concentration of 100 µM. These findings are in line with other cell culture studies, in which reduced cell viability was only observed with concentrations of cis- or oxaliplatin higher than 10 µM [32,33]. In our previous studies we also did not observe apoptotic processes in rat models of cisplatin-induced peripheral neuropathy 14 days after the last administration of the cytostatic drug suggesting CIPN to be rather mediated by functional modulation of DRG neurons than by apoptosis of these cells [16,17]. Furthermore, our former electrophysiological investigations on the function of voltage-gated calcium channels provide evidence for the functionality of DRG neurons treated with different concentrations of cis- or oxaliplatin for 24 h [16,18].

The mitochondrial stress assay showed that DRG neurons demonstrate altered mitochondrial respiration after long-term exposure to cis- or oxaliplatin for 24 h. The lower ATPR and BR possibly determine a diminished ATP generation. The BR indicates an endogenous ATP generation. Consequently, a low BR after exposure to cis- or oxaliplatin indicates a lower ATP generation during the resting state of the DRG neurons, leading to the suggestion that DRG neurons exposed to cis oxaliplatin have a lower ATP generation and ATP supply. However, cisplatin-exposed DRG neurons had lower ATPR and consequently were more affected than oxaliplatin-exposed DRG neurons. Similar results have been shown for the influence of cisplatin on ampullary tissue or cerebellar cortex of guinea pigs [34]. In rat sciatic nerve samples, ATP production deficits were observed after in vivo administration of oxaliplatin or paclitaxel [35].

SRC reflects the ability of neurons to react to different energetic states. Oxaliplatin-exposed DRG neurons demonstrated a lower SRC than cisplatin-exposed DRG neurons, indicating an insufficient energy supply during stressful situations as a contributing mechanism for sensory neuron malfunction or neuronal degeneration.

After platin exposure, a lower MRC and a direct effect on the SRC was observed. Oxaliplatin-exposed DRG neurons showed a lower MRC compared to cisplatin-exposed DRG neurons. These findings pinpoint an inhibitory effect of platin on the mitochondrial respiratory chain complexes I-III as evidenced by a markedly diminished electron flux, which could be chemically restored. The PL of cis- or oxaliplatin-exposed DRG neurons was also reduced. Generally, the PL generates heat and can be influenced by a couple of factors, e.g., the composition of the inner mitochondrial membrane and ROS production. As shown here, both cis- and oxaliplatin exposure increased ROS production, possibly due to changes in the PL, MR, and SRC. In other studies, an increased ROS production after cis- or oxaliplatin exposure has also been observed in Caco-2 and a prostate cancer cell line [36,37].

ROS production of DRG neurons exposed to cisplatin or oxaliplatin showed alterations during long-term and short-term exposure. Cultured DRG neurons treated with cis- or oxaliplatin showed an increased ROS production after 24 h. In recent studies, an increased ROS level of DRG neurons treated with 50 µM cisplatin for 24 h was described [38]. Other studies have shown that DRG neurons exposed to 25 µM oxaliplatin had an increase in ROS production after 24 h [39]. This is in line with the data presented here. We could show an influence of small concentrations of cis- or oxaliplatin on ROS production. In the subset of TRPA1- or TRPV1-positive DRG neurons, an instant increase during cis- or oxaliplatin exposure was observed, when drugs were applied directly to the cells during measurements. Cisplatin-exposed DRG neurons showed a higher ROS production than oxaliplatin-treated cells. The here described findings suggest a different mechanism leading to increased ROS production by long-term or short-term treatment with chemotherapeutics. Here, the short-term effect on ROS production could be directly mediated by chemotherapeutics, while the long-term effect involves further factors such as the release and autocrine effect of cytokines, ATP, or glutamate.

In contrast, we found increased ROS production after exposure with cis- or oxaliplatin, but a decline to or below control level after a short period of time. Possible cyclic regulation of ROS explains the absence of increased ROS production after exposure with 10 µM oxaliplatin after 24 h. The abrupt and significant decline may occur due to antioxidant defenses within the neuron. In a model of diabetic neuropathy, activation of the transcription factor nuclear factor erythroid 2-related factor (Nrf2) in DRG neurons results in an expression of antioxidant enzymes and thus, an immediate decrease in the ROS production and preventing hyperglycemia-induced damage to the neuron [40]. TRPV1-positive subpopulations of DRG neurons were more severely affected after cisplatin exposure than oxaliplatin exposure, resulting in higher ROS production. TRPA1-positive neurons showed a higher ROS production peak (30 min) after exposure to cisplatin. In contrast, exposure with oxaliplatin showed a higher ROS production at the end of the measurements, indicating that TRPA1-positive neurons exhibit a faster antioxidant defense when exposed to cisplatin.

Mitochondrial dysfunction caused by platinum-based chemotherapeutics are well-described for different types of cancer cells. In prostate cancer cell line, exposure with cisplatin for 24 h led to an increase in ROS production [36]. Exposure of human colorectal carcinoma cells with oxaliplatin showed similar effects [41].

The increase in cytosolic calcium concentration and the simultaneous decrease in mitochondrial calcium concentration indicate a discharge of calcium out of the mitochondria into the DRG neurons’ cytosol. In HeLa-S3 cells, exposure with cisplatin led to an increased intracellular calcium concentration. The increase in intracellular calcium was dependent on extracellular calcium concentration. Using an IP3 receptor blocker, the cisplatin-mediated increase of intracellular calcium was diminished [42]. In gastric carcinoma cells, an increased intracellular calcium concentration and IP3 prior to apoptosis was described after cisplatin exposure [43]. Exposure to paclitaxel in a human neuroblastoma cell line induced intracellular calcium oscillations independent of extracellular calcium but dependent on intact signaling via the phosphoinositide signaling pathway [44].

The increased cytosolic calcium concentration during short-term exposure of cisplatin or oxaliplatin contributes to diminished VGCC currents, as observed in a former study [18]. Here, acute exposure of DRG neurons to different oxaliplatin concentrations resulted in reduced VGCC subtype currents. The increased cytosolic calcium concentration after calcium release from mitochondria may alternate the electrochemical gradient for calcium across the biological membrane and contribute to the decrease of VGCC currents [18]. Interestingly, in a study on cisplatin acute application to cultured DRG neurons, L-type, P-/Q-type, and T-type VGCC currents were reduced, while N-type currents were enormously increased [16]. In the current study, we found an increased cytosolic calcium concentration after applying both chemotherapeutics, suggesting an additional mechanism regulating the function of VGCC. An increased cytosolic calcium concentration could signal a change of cis- or oxaliplatin-exposed DRG neurons’ resting membrane potential. A depolarized resting membrane potential of neurons led to hyperexcitability, contributing to neuropathic pain syndromes that often accompany chemotherapy-induced polyneuropathies [45]. Besides the release of calcium from mitochondria, the influx of calcium through VGCC an activation of TRPA1 or TRPV1 by cis- or oxaliplatin via a glutathione-sensitive mechanism could be a further potential mechanism to increase the cytosolic calcium concentration in DRG neurons [46,47,48].

## 4. Materials and Methods

### 4.1. Animals

Adult Wistar rats of both sexes at the age of approximately three weeks (60–80 g) were used to cultivate DRG neurons. The animals were kept at the animal research lab at the University Duisburg-Essen in cages with up to 4 animals. The animals had free access to food and water (ad libitum) and were kept in a 14/10 h day and night rhythm.

All experiments were conducted under the animal welfare guidelines of the University Duisburg-Essen.

### 4.2. Cell Culture of DRG Neurons

For the isolation of the DRG neurons, the rats were narcotized with Isoflurane, and the DRGs were removed as previously described [49]. Briefly, DRGs from all spinal cord segments were removed and placed in ice-cold DMEM/F12 (Dulbecco’s Modified Eagle Medium with F12, Thermo Fisher Scientific, Waltham, MA, USA) solution. Ganglia were prepared from surrounding material to avoid contamination with fibroblasts. Nerve and dorsal roots were removed, capsules were transferred to 1 mg/mL collagenase type II solution and incubated for 45 min at 37 °C and 5% CO_2_. Afterward, DRGs were washed and incubated in 0.25% trypsin/ethylenediaminetetraacetic acid (EDTA) (Thermo Fisher Scientific, Waltham, MA, USA) solution for 8 min at 37° C and 5% CO_2_. The enzyme reaction was stopped with 1 mL of fetal bovine serum (FBS, Thermo Fisher Scientific, Waltham, MA, USA) and washed three times in DMEM/F12. Afterward, the ganglia were titrated in 1 mL of DMEM/F12 until a cell suspension was formed. To facilitate neurons’ adhesion to coverslips, 24-well dishes were placed in an incubator for 2 h. Following the adhesion, 500 µL of DRG-culture medium containing DMEM/F12, 10% fetal bovine serum (FBS, Sigma-Aldrich, Taufkirchen, Germany), 1% penicillin/streptomycin (P/S, Thermo Fisher Scientific, Waltham, MA, USA), 1% neuronal growth factor (NGF, Alomone Labs, Jerusalem, Israel), and 1% N2 (Thermo Fisher Scientific, Waltham, MA, USA) was placed in each well. After 24 h, the medium was replaced with neuronal feeding medium with 10 µM cytosine arabinose (AraC, Thermo Fisher Scientific, Waltham, MA, USA) for 72 h. Seventy-two hours later, the neurons were prepared for further experiments.

### 4.3. Plating Density of DRG Neurons

DRG neurons were counted using a Neubauer Counting Chamber and plated in a density of 1500 neurons/well for all experiments.

### 4.4. Exposure of Sensory Neurons with Cisplatin or Oxaliplatin

Oxaliplatin (Abcam, Cambridge, Great Britain) and cisplatin (Abcam, Cambridge, Great Britain) were diluted with Aqua bidest to a concentration of 10 mM for oxaliplatin and 5 mM for cisplatin as a stock solution. DRG neurons were exposed to 0.1 µM, 1 µM, 10 µM, or 100 µM cisplatin or oxaliplatin for 24 h for measuring viability and ROS production. All further experiments were performed with 10 µM cisplatin or oxaliplatin. For measurement of short-term effect on mitochondrial and cytosolic calcium concentration and ROS production, chemotherapeutics were directly applied to cells during measurements for 70 min. The concentrations used were based on viability experiments and previous cisplatin and oxaliplatin studies [17,18,32,33].

### 4.5. Viability Assay

For examining the influence of cisplatin or oxaliplatin on the viability of cultured DRG neurons, cells were cultured as described above, treated with 0.1 µM, 1 µM, 10 µM, or 100 µM cisplatin or oxaliplatin and a luminescence RealTime-Glo MT Cell Viability Assay (Promega, Walldorf, Germany) was performed according to the manufacture’s protocol.

### 4.6. Immunocytochemical Staining of TRPA1- or TRPV1-Positive DRG Neurons

To determine the effects of the chemotherapeutics on the TRPA1- or TRPV1-positive subpopulations, cell cultures were prepared as described above. The cells were first stained with extracellular TRPA1 or TRPV1 antibodies (Alomone Labs, Jerusalem, Israel).

The cell culture medium was removed from each of the wells and wells were washed twice with cold and sterile blocking solution (2% bovine albumin serum (BSA, Sigma Aldrich, Taufkirchen, Germany) in phosphate-buffered saline (PBS)).

For already tagged primary antibodies (Table 1), cells were incubated for 1 h with the antibody solution at 4 °C, and afterward washed thrice with the ice-cold blocking solution and placed back into the incubator with neuron culture medium.

If a secondary antibody (Table 1) was needed, the primary antibody was incubated at 4 °C for 45 min and washed thrice with cold blocking solution. Afterward, a secondary antibody was added, and the cells were incubated for a further 45 min at 4 °C. The cells were washed three times with blocking solution and likewise returned into the incubator.

### 4.7. ROS Staining

To analyze the long-term effects of cisplatin and oxaliplatin on ROS production in DRG neurons or the short-term effect on TRPA1- or TRPV1-positive subtypes of DRG neurons, cells were cultured and prepared as described above. For the study of short-term effects, DRG neurons were tagged with primary antibodies (Table 1).

For the study of long-term effects, DRG neurons were treated with 0.1 µM, 1 µM, 10 µM, or 100 µM cisplatin or oxaliplatin for 24 h. Afterward, 1 µL of the CellRox (Thermo Fisher Scientific, Waltham, MA, USA) solution was added to 500 µL of the cell culture medium and incubated for 30 min in the incubator. The cells were washed twice with sterile PBS and transferred to the microscopy chamber, and 2 mL of the live-cell imaging solution was added.

For short-term effects, naïve cultured DRG neurons were treated with 10 µM cisplatin or oxaliplatin after CellRox incubation, and images were obtained every 5 min until a 70 min cut-off.

### 4.8. Cytosolic Calcium Concentration

For the determination of the cytosolic calcium concentration of treated TRPA1- or TRPV1-positive DRG neurons, the cells were cultured and stained as described above (Table 1). Cells were stained with primary antibodies as described above (Table 1).

The cells were incubated for 15 min with 0.5 µL Fluo-4 (Thermo Fisher Scientific, Waltham, MA, USA) in 500 µL serum-free culture medium. Afterward, the cells were washed with sterile PBS, 2 mL live-cell imaging solution was added, and cells were treated with 10 µM cisplatin or oxaliplatin. Images were obtained every 5 min until a cut-off at 70 min.

### 4.9. Intramitochondrial Calcium Concentration

To determine the effects of cis- and oxaliplatin on the intramitochondrial calcium concentration, cells were cultured as described above. DRG neurons were stained for TRPA1 or TRPV1, as described above (Table 1).

The cells were incubated for 15 min with 0.5 µL Rhod-2 (Thermo Fisher Scientific, Waltham, MA, USA) in 500 µL serum-free culture medium. Afterward, the cells were washed with sterile PBS, and 2 mL of the live-cell imaging solution was added. The cells were treated with 10 µM cisplatin or oxaliplatin, and images were obtained every 5 min until a 70 min cut-off.

### 4.10. Mitochondrial Stress Assay

The mitochondrial stress assay (SEAHORSE, Agilent, Santa Clara, CA, USA) targets specific complexes of the electron transport chain (ETC) and inhibits them from determining the oxygen consumption rate (OCR). The first inhibitor added is oligomycin inhibits the complex V, the ATP-synthase, of the ETC. The next inhibitor added is carbonyl cyanide-4 (trifluoromethoxy)-phenylhydrazone (FCCP, Agilent, Santa Clara, CA, USA), which collapses the proton gradient and disrupts the membrane potential. The last injection contains a mixture of rotenone, a complex I inhibitor, and Antimycin A, an inhibitor of complex III.

Dorsal root ganglion neurons were cultured as described above and treated with 10 µM cisplatin or oxaliplatin for 24 h.

On the day before the assay, 200 µL of calibrant solution (Agilent, Santa Clara, CA, USA) was added to each well, and the sensor cartridge was lowered until the sensors were submerged into the solution. The sensor cartridge was put in a non-CO_2_ incubator at 37 °C overnight.

On the day of the assay, the cell culture medium was removed and washed once with assay medium before adding 180 µL of assay medium to each well. The injections of the inhibitors were prepared and loaded into the disposable injection ports.

After creating an appropriate assay template with “Wave” software (Agilent, Santa Clara, CA, USA), the sensor cartridge with the hydrated probes was loaded into the Seahorse Analyzer to allow calibration. Afterward, the well-plate containing the cells was put into the Analyzer. Further instructions were followed, as indicated by the apparatus. For analysis of data, we calculated basal respiration (BR), maximal respiratory capacity (MRC), proton leak (PL), ATP-linked respiration (ATPR), spare respiration capacity (SRC), and non-mitochondrial respiration (NMR).

## 5. Conclusions

In summary, our results show a drug-specific effect of cisplatin or oxaliplatin on mitochondrial function in DRG neuron subpopulations. Possibly, mitochondria can be a potential target for future prevention strategies of CIPN.

## Figures and Tables

**Table 1:** Antibodies and dilution used for the TRPA1- and TRPV1-positive staining.

Primary/Secondary Antibody	Dilution, Species	Maker
Anti-TRPA1-ATTO-594	1:200, rabbit	Alomone Labs, Israel
Anti-TRPV1 Alexa Fluo 488	1:200, rabbit	Santa Cruz Biotechnology, USA
Anti-TRPA1 (extracellular)	1:200, rabbit	Alomone Labs, Israel
Anti-TRPV1 (extracellular)	1:200, rabbit	Alomone Labs, Israel
Alexa-Fluo 488	1:200, goat anti-rabbit	DIANOVA, Germany
Cy3	1:200, goat anti-rabbit	DIANOVA, Germany
